# Reforms in County Council Mental Hospitals

**Published:** 1914-01-24

**Authors:** 


### REFORMS IN COUNTY COUNCIL MENTAL HOSPITALS.

The Annual Report of the Asylums Committee of the London County Council states that during the twelve months under review several alterations have been made in the scale of salaries and wages and hours of duty to improve the condition of the service. The Report mentions that consideration has been given to several applications from married attendants who live out of the institutions that they might be allowed to have their meals at home and receive money instead of food. The Committee has not seen its way to consent to these, but has felt that the position of married attendants has deserved sympathetic consideration. For obvious reasons the Committee has not been able to agree to pay higher wages to married than to single men on the ground of marriage alone, but it has adopted a scheme which will benefit the married attendants. This scheme provides that after five years' continuous service every attendant shall be entitled to one stripe, which entitles him to receive a special allowance of £2 10s. a year additional to his pay. At the end of another five years he will have another stripe, also worth an extra £2 10s. a year. Dealing generally with the question of married attendants, etc., the Report says:

"Hitherto non-resident head attendants, attendants and boarded servants, and resident married attendants have been entitled to draw a money allowance in lieu of their rations on their whole day of leave once a week. This privilege, however, has not applied to the great majority of the resident boarded staff. Recognising that the emolument of board is really a part of the employee's wage, valued as such at 10s. a week, or 1s. 5d. a day, we have felt that all members of the fully boarded subordinate staff, both male and female, and whether resident or not, are in equity entitled to receive a money payment in lieu of their meals, and . . . we have, therefore, amended our rule as to the allowance of money in lieu of rations during absence on occasional leave to provide for such a payment to be made."

Provision has existed for some time that the weekly leave of nurses and attendants may, if convenient to the administration, accumulate for two weeks once a quarter, and that an extra night's leave may then be granted so that the employees may have an opportunity of absenting themselves for two clear days from the institution. In the case of attendants this privilege has been extended so that the accumulation may now take place once in six weeks. Most significant of all, however, is the following paragraph: "Hitherto our general rules have provided that of the assistant medical officers employed at each asylum only the first may be married. With the consent of the Secretary of State to the necessary amendment of the rules we have now arranged that the second assistant medical officer may also, if he desires, be a married man."

This provides the latest instance of the movement now publicly on foot to improve the status and the prospects of the assistant medical officer in mental hospitals. It is also only another example of the at first sight curious fact that such improvement as is accorded to the junior medical officer is but a levelling up of his position in certain respects to that already granted to the attendants. We have previously pointed out that the difference between these two sections of the medical and nursing staff has largely to be explained by the co-operation which has till lately been the peculiar characteristic of the latter alone. Opinion, however, is at present divided among medical officers as to how much may be expected to be gained by combination. While the value of the principle is sufficiently attested by experience, many men hold that reform is more to be expected from the general pressure of a bad system which is making it increasingly difficult to retain, and, hence, even to attract, the services of the right class of man for resident posts in mental hospitals. It is pointed out that the voluntary hospitals have been obliged to raise the salaries of their resident medical officers from this cause, without the invocation of any form of combination, and though the positions of the voluntary hospitals and the public mental hospitals differ in many important respects, it seems fair to argue that once the necessity of raising salaries is generally admitted, the desired privileges which an increased income makes financially possible, such as permission to marry, must soon be recognised as a necessity also. We therefore welcome the above reforms, not as affecting the argument for combination, but as showing that at last the need for granting additional privileges to medical officers in mental hospitals, of which the movement for combination is a symptom, is receiving a slow but steady recognition by those who control the amenities of the service.

